# Self-harm, suicidal ideation, and the positive symptoms of psychosis: Cross-sectional and prospective data from a national household survey

**DOI:** 10.1016/j.schres.2021.06.021

**Published:** 2021-07

**Authors:** Angharad N. de Cates, Gennaro Catone, Steven Marwaha, Paul Bebbington, Clara S. Humpston, Matthew R. Broome

**Affiliations:** a Department of Psychiatry, University of Oxford, Oxford, UK; b Oxford Health NHS Foundation Trust, Oxford, UK; c Department of Educational, Psychological and Communication Sciences, Suor Orsola Benincasa University, Naples, Italy; d Institute for Mental Health, School of Psychology, College of Life and Environmental Sciences, University of Birmingham, Birmingham, UK; e Division of Psychiatry, University College London, London, UK; f Birmingham Women's and Children's NHS Foundation Trust, UK

**Keywords:** Schizophrenia, Psychosis, Epidemiology, Self-harm, Suicide

## Abstract

**Background:**

Schizophrenia is associated with premature mortality, partly through increased suicide rates.

**Aims:**

To examine (1) if persecutory ideas, auditory hallucinations, and probable cases of psychosis are associated with suicidal thoughts or attempts cross-sectionally and prospectively, and (2) if such links are mediated by specific affective factors (depression, impulsivity, mood instability).

**Method:**

We analysed the 2000, 2007, and 2014 British Adult Psychiatric Morbidity Surveys (APMS) separately. Measures of psychosis provided independent variables for multi-stage logistic regressions, with suicidal thoughts and attempts as dependent variables. We also conducted analyses to assess mediation by affective variables, and longitudinal analyses on a subset of the 2000 dataset.

**Results:**

In every dataset, persecutory ideas, auditory hallucinations and probable psychosis were associated cross-sectionally with lifetime suicidal attempts and thoughts, even after controlling for confounders, with a single exception (persecutory ideation and suicide attempts were unconnected in APMS 2014). Cross-sectional associations between auditory hallucinations and suicidal phenomena were moderated by persecutory ideation. In the 2000 follow-up, initial persecutory ideas were associated with later suicidal thoughts (O.R. 1.77, *p* < 0.05); there were no other longitudinal associations. In the 2007 and 2014 datasets, mood instability mediated the effects of psychotic phenomena on suicidality more strongly than impulsivity; depression was also an important mediator. There were appreciable direct effects of positive symptoms on suicidal thoughts and behaviour.

**Conclusions:**

Improving psychotic symptoms and ameliorating co-morbid distress may in itself be effective in reducing suicidal risk in schizophrenia. Given their potential mediating role, mood instability and depression may also be targets for intervention.

## 1 Introduction

On average people with schizophrenia spectrum psychosis die 15–20 years earlier than the rest of the general population (Reininghaus et al., 2015). Although smoking and cardiometabolic risk factors disproportionately affect those with psychosis increasing the risk of natural causes of death, suicide rates among people with psychosis are also significantly greater than the general population (Yung et al., 2020). One in every 20 individuals with schizophrenia will die by suicide (Palmer et al., 2005), twenty times the United Kingdom (UK) national rate (Reininghaus et al., 2015), and equivalent to 4.3 deaths per 1000 person-years (Bjorkenstam et al., 2014). Non-fatal self-harm is also common in people with schizophrenia, with a reported lifetime prevalence of 39%, compared to 3–7% in unaffected individuals (Fuller-Thomson and Hollister, 2016; Mcmanus et al., 2016). In people with schizophrenia spectrum psychosis, suicidal ideation is strongly associated with suicide over the next decade (Chapman et al., 2015), and those with current psychotic symptoms (even when these are subclinical) appear to be at increased risk of self-harm thoughts, acts and suicide (Kelleher et al., 2017; Koyanagi et al., 2015; Yates et al., 2019). This indicates an opportunity for clinicians to intervene to reduce levels of self harm and suicide. Greater understanding of what leads to suicidality is critical to this strategy.

Suicide has been associated with a range of symptoms in schizophrenia, namely suspiciousness, persecutory delusions, agitation, negative symptoms, depression and hopelessness, and command hallucinations (Ventriglio et al., 2016). However, we currently lack information about the relative impact of different positive symptoms on the risk of self-harm. In the general population, mood instability appears to be associated with suicidal ideation and behaviour in the context of poor sleep (McDonald et al., 2017). Mood instability is also important in emerging and ongoing psychotic symptoms (Marwaha et al., 2014; Palmier-Claus et al., 2012), but its role in relation to self-harm in psychosis is less clear. Such self-harm has also been linked to impulsivity, which may also be a mediator of longer-term clinical outcome (Nanda et al., 2016). Mood instability and impulsivity have a complex relationship, particularly as potential mediators of self-harm (de Cates et al., 2019). Previous work using community data has identified that psychotic symptoms are associated with self-harm risk, and that this relationship is not necessarily explained by symptoms of borderline personality disorder (which include impulsivity and mood instability) (Kelleher et al., 2017). Others indicate that impulsivity may be redundant as a risk for future suicidal thoughts once mood instability is accounted for (Peters et al., 2016).

Most studies of the relationship between self-harm and schizophrenia spectrum psychosis have used hospital samples (Altamura et al., 2003; Barak et al., 2008; Gut-Fayand et al., 2001; Roy, 2005), whereas most people with schizophrenia reside in the community. Community samples have been analysed in Denmark (Osler et al., 2015), Sweden (Fazel et al., 2014), and Canada (Fuller-Thomson and Hollister, 2016), but few within the UK. In the general population, the association between psychotic experiences, and suicidal thoughts and behaviours, is robust even after accounting for potential confounders (Hielscher et al., 2018), increasing the risk of self-harm approximately threefold (DeVylder et al., 2015; Honings et al., 2016). In one community study of people with non-affective psychosis, three quarters had suicidal ideation, and 4.5% had attempted suicide in the preceding four weeks (Freeman et al., 2019). Suicidal thoughts were associated with higher levels of depression, persecutory thoughts, hallucinations, poor sleep, anger and negative cognitions. Psychotic experiences are thus a risk marker for suicidal thoughts and behaviours (Hielscher et al., 2018). Self-harm episodes of all forms are associated with future attempts and completed suicides (Barak et al., 2008; Hawton et al., 2005).

In the current analysis we use large community samples to establish the cross-sectional and longitudinal association of psychosis (at a symptomatic level equivalent to diagnosis of mental illness) and specific psychotic symptoms with self-harm and suicidal thoughts. We also explored the potential role of mediating factors. We tested three hypotheses:-.

1. Persecutory ideas, auditory hallucinations, and ‘probable psychosis’ are associated with suicidal thoughts and attempts cross-sectionally
2. These psychotic phenomena are linked prospectively to the persistence, repetition and inception of suicial thoughts and acts
3. The link between psychotic and suicidal phenomena is mediated by depression, impulsivity and mood instability

## 2 Materials and methods

### 2.1 Samples

We used data from British *Adult Psychiatric Morbidity Surveys (APMS)*. These provide national statistics for mental illness and treatment access in the household population. For the three surveys the numbers of respondents were as follows: 2000 survey - 8580; 2007 survey - 7403; 2014 survey – 7546. The age ranges included varied between datatsets: all surveys had a minimum age of 16, but the 2000 data had a maximum age of 74 whereas in 2007 and 2014 there was no maximum age limit. Mean ages across the datasets were: 45.4, SD 15.6 (2000); 51.1, SD 18.6 (2007); 52.3, SD 18.8 (2014). Response rates were 69% in 2000, and 57% in 2007 and in 2014.

The measures were the same in all datasets. The 2000 dataset included a sub–sample of 2406 participants followed up after 18 months. Additional information regarding selection, sampling and assessment can be found in the relevant survey reports (Byron et al., 2016; McManus et al., 2009; Singleton et al., 2001; Singleton and Lewis, 2003). Data were weighted to provide analyses representative of the household population. Sample characteristics are given in supplementary Table 1. Across the three datasets, there are random fluctuations in the numbers of participants endorsing each variable; there is a significant fluctuation for the means of IQ (F(2,23526) = 146.567, *p* < 0.0001). We used the 2000, 2007, and 2014 surveys for the first aim, the 2000 dataset with its 18 month follow-up for the second, and the 2007 and 2014 surveys for the remaining analyses.

### 2.2 Variables

#### 2.2.1 Psychotic variables

The Psychotic Screening Questionnaire (PSQ) (Bebbington & Nayani, 1995) was used in each survey. Persecutory ideation was determined from the question *“Over the past year have you felt that people were deliberately acting to harm you/your interests?” (PSQ3a)*. Auditory hallucinations required a positive answer to *“In the past year have you heard voices saying quite a few words or sentences?”* (PSQ5a). In the longitudinal analyses we also used the broader, screening, questions for psychotic symptoms: “*Have you felt that people were against you in past year?*” (PSQ3) and “*Have you heard/seen things that other people could not?*” (PSQ5). The category “probable psychosis” (implying a likely clinical diagnosis) was applied if 1) the respondent was given a diagnosis of psychosis in the phase two interview using the *Schedules for Clinical Assessment in Neuropsychiatry* (SCAN) (Wing et al., 1990); or 2) met at least two of the following screening criteria: current antipsychotic drug medication; an inpatient stay for a emotional or mental disorder in the past 3 months or admission to a ward or hospital specialising in mental health problems at any time; a positive response to the question PSQ5a (auditory hallucinations); and a self-reported diagnosis of psychotic disorder or of symptoms suggestive of such a disorder. For those who screened negative for psychotic disorder using screening criteria, it was assumed that these were true negatives regardless of whether or not a SCAN assessment was completed. For those who screened positive for psychotic disorder on screening questions and had a SCAN assessment, the results of the SCAN were used. This framework has been used in previous research (Catone et al., 2015; Marwaha et al., 2014; Marwaha et al., 2015). All variables were dichotomous.

#### 2.2.2 Suicidal attempts/thoughts

All surveys used items from the Clinical Interview Schedule Revised (CIS-R) to record the presence of suicidal thoughts and suicide attempts over the life course (i.e. *“Thought about suicide ever”; “Attempted suicide ever”*). In the 2000 follow-up, suicide attempts and thoughts since the first interview were recorded.

#### 2.2.3 Sexual abuse

For APMS2000 and 2007, participants were shown a card listing stressful events and asked to indicate which they had experienced at some time in their lives. The list included sexual abuse, as analysed here. For APMS2014, we used the corresponding TRAUMA variable: *“Experienced sexual abuse at any time in your life?”*

#### 2.2.4 Impulsivity, mood instability and depression

The presence of impulsivity and mood instability was established from questions in relation to “the last several years” in the Structured Clinical Interview for DSM-IV (SCID-II) Personality Disorder (First et al., 1997). Depressed mood was derived from 4 linked CIS-R questions (*“Have you enjoyed or taken an interest in things as much as usual in past week?”; “How many days have you felt depressed in past week?”; “Have you felt depressed for more than 3 hours on any day in past week?”; “Have you ever become happier when something nice happened or when in company in past week?”*), thereby creating a 5-level ordinal variable.

#### 2.2.5 Drug misuse and alcohol problems

Drug misuse was assessed by asking respondents if they had ever used any of a list of street drugs. Hazardous alcohol use was defined as a score of 8 or more on the Alcohol Use Disorders Identification Test (Saunders et al., 1993).

#### 2.2.6 Sociodemographic variables

We included *sex*, *marital status* (married/not married), *employment status* (employed/not employed), *ethnicity* (others/black) *age band* (16–24/25–34/35–44/45–54/55–64/65–74), *IQ* (*2000*: 10–89/90–109/110–129; *2007 & 2014*: 70–85/86–100/101+).

### 2.3 Statistical analyses

These were completed using the Statistical Package for Social Sciences - 20 (SPSS 20th edition for Windows), R (version 3.6.1 in a Windows environment, packages ‘gmodels’, ‘stats’ and ‘car’) and STATA (version 14 for Windows). SPSS was used to prepare the data for analysis and descriptive statistics. R and STATA were used for all other analyses. No manipulation was required to account for missing data within the dataset.

#### 2.3.1 APMS2000/2007/2014 cross-sectional analyses

We conducted multi-stage logistic regressions with a) persecutory ideas, b) auditory hallucinations, and c) probable psychosis as independent variables (independent variables (IVs)) and i) suicidal attempts and ii) suicidal thoughts as dependent variables (dependent variables (DVs)), using each dataset separately. Regressions were initially conducted unadjusted, followed by the sequential inclusion of each group of confounders. This resulted in five regression levels 1: unadjusted, 2: as previous plus socio-demographic information (sex, age, marital status, employment, ethnicity, IQ), 3: as previous plus stressful events (sexual abuse), 4: as previous plus affective variables (mood instability, depression), 5: as previous plus drug and alcohol problems.

#### 2.3.2 APMS2000 longitudinal analyses

The same regression model was used as above, with the exception that we omitted the probable psychosis variable. Two designs were analysed: 1) the continuation test (when the dependent variable was recognized both at T1 and T2); and 2) the new inception test (when the dependent variable was present only at T2).

#### 2.3.3 APMS2000/2007/2014 multiple regression analyses

To establish which symptoms were most strongly associated with suicidal ideation and self-harm cross-sectionally, we entered auditory hallucinations and persecutory ideation as independent variables in multi-stage logistic regressions, using each dataset in turn. The dependent variables comprised suicidal attempts and suicidal thoughts.

For all the multiple logistic regression analyses we used the more closely specified psychotic variables (PSQ3a and PSQ5a) as they provide more securely clinical implications. However, in the longitudinal analysis we also present results for their less specific counterparts (PSQ3 and PSQ5). Cross-sectional (multistage) logistic regression involves each independent variable individually (persecutory ideas; auditory hallucinations; probable psychosis). Cross-sectional (multistage) multiple regression allows separate independent variables to be presented together to allow exploration of the potential interaction. To increase transparency, we have presented findings from both methods.

#### 2.3.4 APMS2007 mediation analyses

We used the Karlson Holm Breen (KHB) test (Linden and Karlson, 2013) to explore the role of mediators in the association between psychotic symptoms (persecutory ideas and auditory hallucinations) and suicidal attempts/thoughts. This decomposes the coefficient into *total*, *direct* and *indirect* effects. The putative mediators were initially introduced together and then separately. In this way, it is possible to analyse both their combined and individual effects, as in other analyses (Marwaha et al., 2014; Moffa et al., 2017). We adjusted the analyses for potential confounders (sex and age).

## 3 Results

### 3.1 Cross-sectional associations between psychotic measures and suicidal thoughts/attempts

Persecutory ideation (PSQ3a) was strongly associated cross-sectionally with suicidal thoughts and attempts in both the 2000 and 2007 surveys, even after controlling for all confounders. These associations remained for the 2014 dataset, although only at borderline significance for suicidal attempts when all counfounders were included. In the 2000 dataset, persecutory ideation was significantly associated with both suicidal thoughts (OR 2.43 *p* < 0.001) and attempts (OR 1.94 *p* < 0.001). In the 2007 dataset, the equivalent ORs were 3.06 (*p* < 0.001) and 2.60 (*p* < 0.001), and in the 2014 dataset OR 1.66 (p < 0.001), and 1.37 (*p* > 0.05).

After controlling for confounders, auditory hallucinations (PSQ5a) were significantly associated with both suicidal thoughts and attempts in all datasets. For suicidal thoughts, the odds ratios were 2.52 (*p* < 0.05) in APMS2000, 6.72 (*p* < 0.001) in AMPS2007, and 2.53 (p < 0.001) in APMS2014. The corresponding odds ratios for suicidal attempts were APMS2000: 3.54 (*p* < 0.01), APMS2007: 2.71 (*p* < 0.05), APMS2014: 2.28 (p < 0.01).

After controlling for confounders, probable psychosis was particularly strongly linked to suicide attempts in the 2000 and 2007 datasets (APMS2000: OR 9.86 *p* < 0.001; APMS2007: OR 7.63 *p* < 0.001). The corresponding odds ratio for the 2014 dataset was somewhat smaller (3.00 *p* < 0.01). The association of probable psychosis with suicidal thoughts was weaker, though still strongly significant in the 2000 and 2007 datasets (APMS2000: OR 3.64 *p* < 0.01; APMS2007: OR 4.97 *p* < 0.01; APMS2014: OR 2.56 p < 0.05). These results are given in more detail in Table 1a, Table 1b, Table 1c.

**Table 1a:** 2000, 2007 and 2014 Cross-sectional multi-stage logistic regression analyses between persecutory ideas (IV) and 1) suicide attempts and 2) suicide thoughts (DVs).

Persecutory ideas	Suicide attempts	Suicide thoughts
	OR (CI) p *(<0.001), ** (<0.01), # (<0.05)	
2000		
Unadjusted	5.01 (3.94–6.38)*	4.71 (4.01–5.54)*
Controlled for socio-demographics^a^	4.09 (3.15–5.33)*	4.28 (3.56–5.14)*
Controlled for above+stressful events^b^	3.33 (2.52–4.41)*	3.82 (3.15–4.64)*
Controlled for above+affective traits^c^	2.14 (1.58–2.88)*	2.59 (2.11–3.19)*
Controlled for above+drug and alcohol misuse	1.94 (1.43–2.63)*	2.43 (1.97–3.00)*

2007		
Unadjusted	6.42 (4.84–8.52)*	6.27 (5.15–7.64)*
Controlled for socio-demographics^a^	5.14 (3.77–7.01)*	5.39 (4.34–6.70)*
Controlled for above+stressful events^b^	4.39 (3.16–6.12)*	4.95 (3.95–6.20)*
Controlled for above+affective traits^c^	2.63 (1.85–3.75)*	3.10 (2.41–3.99)*
Controlled for above+drug and alcohol misuse	2.60 (1.83–3.69)*	3.06 (2.39–3.92)*

2014		
Unadjusted	3.12 (2.43–3.97)*	3.14 (2.65–3.70)*
Controlled for socio-demographics^a^	2.50 (1.91–3.23)*	2.69 (2.26–3.21)*
Controlled for above+stressful events^b^	2.24 (1.70–2.94)*	2.58 (2.15–3.09)*
Controlled for above+affective traits^c^	1.38 (1.00–1.89)#	1.75 (1.39–2.18)*
Controlled for above+drug and alcohol misuse	1.37 (0.99–1.88)	1.66 (1.32–2.08)*

a Marital status, sex, age, employment status, ethnicity, IQ.

b Sexual abuse.

c Depression, mood instability.

**Table 1b:** 2000, 2007 and 2014 Cross-sectional multi-stage logistic regression analyses between auditory hallucinations (IV) and 1) suicide attempts and 2) suicide thoughts (DVs).

Auditory hallucinations	Suicide attempts	Suicide thoughts
	OR (CI) p *(<0.001), ** (<0.01), # (<0.05)	
2000		
Unadjusted	10.66 (6.37–17.86)*	6.62 (4.12–10.65)*
Controlled for socio-demographics^a^	8.36 (4.71–14.83)*	5.75 (3.29–10.04)*
Controlled for above+stressful events^b^	5.78 (3.10–10.78)*	4.32 (2.25–8.32)*
Controlled for above+affective traits^c^	3.75 (1.90–7.37)*	2.65 (1.27–5.53)**
Controlled for above+drug and alcohol misuse	3.54 (1.71–7.32)**	2.52 (1.13–5.64)#

2007		
Unadjusted	11.59 (6.42–20.92)*	13.63 (7.47–24.88)*
Controlled for socio-demographics^a^	6.85 (3.43–13.69)*	11.81 (5.65–24.69)*
Controlled for above+stressful events^b^	5.03 (2.41–10.49)*	10.44 (4.84–22.48)*
Controlled for above+affective traits^c^	2.78 (1.13–6.83)#	7.00 (2.80–17.50)*
Controlled for above+drug and alcohol misuse	2.71 (1.12–6.59)#	6.72 (2.83–15.98)*

2014		
Unadjusted	4.32 (3.01–6.07)*	3.35 (2.54–4.38)*
Controlled for socio-demographics^a^	3.23 (2.16–4.71)*	2.99 (2.23–3.99)*
Controlled for above+stressful events^b^	2.73 (1.77–4.08)*	2.72 (2.00–3.67)*
Controlled for above+affective traits^c^	2.20 (1.33–3.52)*	2.46 (1.67–3.60)*
Controlled for above+drug and alcohol misuse	2.28 (1.37–3.69)**	2.53 (1.70–3.74)*

a Marital status, sex, age, employment status,ethnicity, IQ.

b Sexual abuse.

c Depression, mood instability.

**Table 1c:** 2000, 2007 and 2014 Cross-sectional multi-stage logistic regression analyses between probable psychosis (IV) and 1) suicide attempts and 2) suicide thoughts (DVs).

Probable psychosis	Suicide attempts	Suicide thoughts
	OR (CI) p *(<0.001), ** (<0.01), # (<0.05)	
2000		
Unadjusted	27.39 (14.89–50.40)*	14.57 (8.05–26.38)*
Controlled for socio-demographics^a^	19.16 (9.30–39.45)*	8.71 (4.14–18.31)*
Controlled for above+stressful events^b^	12.62 (6.12–26.01)*	6.00 (2.76–13.05)*
Controlled for above+affective traits^c^	8.90 (3.85–20.54)*	3.47 (1.38–6.73)**
Controlled for above+drug and alcohol misuse	9.86 (4.17–23.29)*	3.64 (1.39–9.51)**

2007		
Unadjusted	34.17 (17.75–65.76)*	21.23 (9.40–47.93)*
Controlled for socio-demographics^a^	18.78 (8.88–39.73)*	13.19 (5.51–31.55)*
Controlled for above+stressful events^b^	16.44 (7.11–38.02)*	11.73 (4.73–29.06)*
Controlled for above+affective traits^c^	8.23 (3.10–21.86)*	5.40 (1.84–15.83)**
Controlled for above+drug and alcohol misuse	7.63 (2.87–20.25)*	4.97 (1.64–15.02)**

2014		
Unadjusted	22.53 (14.17–35.98)*	15.11 (9.14–26.10)*
Controlled for socio-demographics^a^	13.25 (8.07–21.84)*	11.74 (6.96–20.67)*
Controlled for above+stressful events^b^	7.70 (4.45–13.32)*	8.17 (4.68–14.80)*
Controlled for above+affective traits^c^	2.91 (1.47–5.78)**	2.51 (1.14–5.92)#
Controlled for above+drug and alcohol misuse	3.00 (1.50–6.04)**	2.56 (1.15–6.12)#

a Marital status, sex, age, employment status,ethnicity, IQ.

b Sexual abuse.

c Depression, mood instability.

### 3.2 Longitudinal associations between psychotic symptoms and suicidal thoughts/attempts

Based on the broader psychotic variables PSQ3 and PSQ5 in the APMS2000 18-month follow-up analyses, initial persecutory ideas were associated with the *persistence* of suicidal thoughts even after controlling for all confounders (O.R. 1.77, *p* < 0.05). Persecutory ideation also was longitudinally associated with the de novo emergence of suicidal thoughts at the unadjusted level (O.R. 2.24); there was little reduction in the size of this association when we controlled for affective traits and drug and alcohol abuse, though it fell short of significance (O.R. 1.64, *p* > 0.05) (see Table 2a, Table 2b). None of the remaining analyses yielded significant results, including all those involving hallucinosis.

**Table 2a:** 2000 FUP analyses between persecutory symptoms and auditory/visual hallucinations (IVs) and suicide attempts and thoughts (DV) – repetition.

Repetition (T1 + T2)		
Persecutory ideation (psq3)	Suicide attempt	Suicidal thoughts
	OR (CI) p *(<0.001), ** (<0.01), # (<0.05)	
Unadjusted	2.62 (0.61–11.23)	2.20 (1.43–3.39)*
Controlled for sociodemographics^a^	4.48 (0.70–28.67)	2.05 (1.29–3.25)**
Controlled for above+stressful events^b^	5.61 (0.88–35.57)	1.95 (1.22–3.11)**
Controlled for above+affective traits^c^	2.56 (0.25–26.03)	1.78 (1.10–2.89)#
Controlled for above+drug and alcohol misuse	2.62 (0.24–29.95)	1.77 (1.09–2.88)#


Repetition (T1 + T2)		
Auditory/vhallucinations (psq5)	Suicidal attempts	Suicidal thoughts
Unadjusted	3.18 (0.74–13.70)	1.37 (0.74–2.54)
Controlled for sociodemographics^a^	2.52 (0.33–19.35)	1.15 (0.56–2.38)
Controlling for above+stressfulevents^b^	3.91 (0.44–34.40)	1.10 (0.53–2.27)
Controlling for above+affectivetraits^c^	4.70 (0.51–42.97)	1.27 (0.63–2.56)
Controlling for above+drug and alcohol misuse	5.75 (0.59–55.79)	1.26 (0.63–2.50)

a Marital status, sex, age, employment status, ethnicity, IQ.

b Sexual abuse.

c Depression, mood instability.

**Table 2b:** 2000 FUP analyses between persecutory ideas and auditory/visual hallucinations (IVs) and suicide attempts and thoughts (DV) – new inception.

New inception (T2)		
Persecutory ideas (psq3)	Suicide attempt	Suicidal thoughts
	OR (CI) p *(<0.001), ** (<0.01), # (<0.05)	
Unadjusted	0.69 (0.15–3.14)	2.24 (1.28–3.92)**
Controlled for sociodemographics^a^	0.58 (0.10–3.25)	1.99 (1.03–3.85)#
Controlled for above+stressfulevents^b^	0.62 (0.11–3.40)	2.03 (1.08–3.72)#
Controlled for above+affectivetraits^c^	0.42 (0.07–2.53)	1.56 (0.84–2.91)
Controlled for above+drug and alcohol misuse	0.46 (0.06–3.17)	1.64 (0.88–3.04)


New inception (T2)		
Auditory/visual hallucinations (psq5)	Suicidal attempts	Suicidal thoughts
Unadjusted	3.25 (0.58–18.03)	1.45 (0.53–3.94)
Controlled for sociodemographics^a^	2.25 (0.35–14.27)	1.27 (0.44–3.63)
Controlled for above+stressfulevents^b^	2.21 (0.34–14.02)	1.29(0.45–3.70)
Controlled for above+affectivetraits^c^	1.78 (0.32–9.82)	0.97 (0.34–2.77)
Controlled for above+drug and alcohol misuse	1.97 (0.34–11.38)	1.04 (0.36–3.01)

a: marital status, sex, age, employment status,ethnicity, IQ; b: sexual abuse; c: depression, mood instability.

Using the narrow psychotic variables PSQ3a and PSQ5a in the same follow-up analyses (see Table 3a, Table 3b), the unadjusted association of persecutory ideas with suicidal thoughts was significant (OR 1.96 *p* < 0.01); this association became non-significant after controlling for our chosen confounders (OR 1.63). A similar picture was seen for auditory hallucinations and suicidal thoughts. No other results were significant. In contrast, analyses involving auditory hallucinations and suicide attempts were unstable, with large confidence intervals. We presume these unstable results are related to the small numbers of those reporting suicide attempts at T2 and a lack of precision in the data due to the more specific nature of these variables.

**Table 3a:** 2000 FUP analyses between persecutory symptoms and auditory hallucinations (IVs) and suicide attempts and thoughts (DV) – repetition.

Repetition (T1 + T2)		
Persecutory ideation (psq3a)	Suicide attempts	Suicide thoughts
	OR (CI) p *(<0.001), ** (<0.01), # (<0.05)	
Unadjusted	2.20 (0.60–8.01)	1.96 (1.27–3.01)**
Controlled for sociodemographics^a^	2.30 (0.39–13.39)	1.94 (1.23–3.06)**
Controlled for above+stressful events^b^	2.45 (0.41–14.53)	1.82 (1.14–2.90)#
Controlled for above+affective traits^c^	1.06 (0.24–4.65)	1.64 (1.01–2.66)#
Controlled for above+drug and alcohol misuse	1.06 (0.29–3.90)	1.63 (0.99–2.68)


Repetition (T1 + T2)	
Auditory hallucinations (psq5a)	Suicide thoughts
Unadjusted	3.62 (1.13–11.63)#
Controlled for sociodemographics^a^	4.47 (1.13–17.63)#
Controlling for above+stressful events^b^	3.62 (0.84–15.47)
Controlling for above+affective traits^c^	3.74 (1.13–12.40)#
Controlling for above+drug and alcohol misuse	3.72 (1.12–12.37)#

**Table 3b:** 2000 FUP analyses between persecutory symptoms and auditory hallucinations (IVs) and suicide attempts and thoughts (DV) – new inception.

New inception (T2)		
Persecutory ideas (psq3a)	Suicide attempts	Suicide thoughts
	OR (CI) p *(<0.001), ** (<0.01), # (<0.05)	
Unadjusted	1.29 (0.23–7.07)	2.16 (1.04–4.48)#
Controlled for sociodemographics^a^	1.15 (0.17–7.62)	2.22 (1.03–4.80)#
Controlled for above+stressfulevents^b^	1.26 (0.18–8.57)	2.29 (1.05–4.97)#
Controlled for above+affectivetraits^c^	1.05 (0.13–8.17)	1.93 (0.88–4.26)
Controlled for above+drug and alcohol misuse	1.02 (0.09–11.18)	1.96 (0.89–4.35)


New inception (T2)	
Auditory hallucinations (psq5a)	Suicide thoughts
Unadjusted	1.95 (0.24–15.56)
Controlled for sociodemographics^a^	1.66 (0.20–13.62)
Controlled for above+stressful events^b^	1.69 (0.20–13.76)
Controlled for above+affective traits^c^	1.24 (0.18–8.45)
Controlled for above+drug and alcohol misuse	1.34 (0.19–9.31)

### 3.3 Multiple regression analyses combining persecutory ideation and auditory hallucinations in relation to suicidal phenomena

Table 4 shows the broadly similar results of multiple logistic regression analyses using the narrower variables PSQ3a and 5a. In the 2000 dataset, both persecutory ideation and auditory hallucinations were independently linked to *suicide attempts* after controlling for confounders. While persecutory ideation was linked to *suicidal thoughts*, auditory hallucinations were not, although the odds ratios were similar. Based on the 2007 data, suicidal thoughts and attempts both showed similar significant and sizeable associations with persecutory ideation. The findings for auditory hallucination in the 2007 dataset differed from those in 2000: the association of auditory hallucinations with suicidal thoughts was significant, while that with suicide attempts was not. All associations of suicidal phenomena with psychotic symptoms were significant in the 2014 dataset.

**Table 4:** 2000, 2007 and 2014 Cross-sectional multi-stage multiple regression analyses between persecutory ideas (psq3a) and auditory hallucinations (psq5a) (IVs) and 1) suicide attempts and 2) suicide thoughts (DVs).

	Suicide attempts	Suicide thoughts
	OR (CI) p *(<0.001), ** (<0.01), # (<0.05)	
2000		
Unadjusted		
Psq3a	4.55 (3.55–5.85)*	4.50 (3.81–5.30)*
Psq5a	6.57 (3.68–11.72)*	4.38 (2.52–7.60)*
Controlled for socio-demographics^a^		
Psq3a	3.82 (2.92–5.00)*	4.12 (3.42–4.97)*
Psq5a	5.94 (3.26–10.82)*	4.01 (2.13–7.54)*
Controlled for above+stressful events^b^		
Psq3a	3.18 (2.39–4.22)*	3.73 (3.06–4.54)*
Psq5a	4.54 (2.40–8.58)*	3.22 (1.55–6.70)**
Controlled for above+affective traits^c^		
Psq3a	2.07 (1.53–2.80)*	2.55(2.07–3.15)*
Psq5a	3.34 (1.70–6.54)*	2.20 (1.01–4.78)#
Controlled for above+drug and alcohol misuse		
Psq3a	1.88 (1.39–2.57)*	2.39 (1.94–2.96)*
Psq5a	3.24 (1.58–6.63)**	2.11 (0.92–4.86)

2007		
Unadjusted		
Psq3a	5.79 (4.32–7.76)*	5.88 (4.82–7.17)*
Psq5a	6.33 (3.05–13.10)*	9.03 (4.54–17.95)*
Controller for socio-demographics^a^		
Psq3a	4.86 (3.55–6.66)*	5.13 (4.13–6.38)*
Psq5a	4.56 (2.12–9.81)*	8.29 (3.83–17.95)*
Controlled for above+stressful events^b^		
Psq3a	4.21 (3.02–5.88)*	4.73 (3.77–5.93)*
Psq5a	3.55 (1.65–7.63)**	7.33 (3.33–16.13)*
Controlled for above+affective traits^c^		
Psq3a	2.58 (1.80–3.69)*	3.02 (2.34–3.88)*
Psq5a	2.38 (0.93–3.69)	5.86 (2.34–14.66)*
Controlled for above+drug and alcohol misuse		
Psq3a	2.54 (1.78–3.63)*	2.96 (2.31–3.80)*
Psq5a	2.24 (0.89–5.65)	5.59 (2.27–13.77)*
2014		
Unadjusted		
Psq3a	2.99 (2.33–3.82)*	3.08 (2.60–3.64)*
Psq5a	4.01 (2.78–5.66)*	3.17 (2.40–4.18)*
Controlled for socio-demographics^a^		
Psq3a	2.47 (1.89–3.18)*	2.66 (2.22–3.17)*
Psq5a	3.14 (2.10–4.60)*	2.89 (2.25–3.87)*
Controlled for above+stressful events^b^		
Psq3a	2.23 (1.69–2.92)*	2.55 (2.12–3.05)*
Psq5a	2.69 (1.75–4.03)*	2.63 (1.93–3.56)*
Controlled for above+affective traits^c^		
Psq3a	1.40 (1.01–1.91)#	1.75 (1.39–2.19)*
Psq5a	2.22 (1.35–3.55)**	2.45 (1.67–3.59)*
Controlled for above+drug and alcohol misuse		
Psq3a	1.38 (1.00–1.89)#	1.66 (1.32–2.09)*
Psq5a	2.30 (1.39–3.70)**	2.53 (1.70–3.73)*

a Marital status, sex, age, employment status, ethnicity, IQ.

b Sexual abuse.

c Depression, mood instability.

### 3.4 Mediation analyses

In the logistic regression model linking *persecutory ideation* with suicidal phenomena derived from the APMS2007 dataset both the direct and indirect coefficients were positive and significant (Table 2a in supplementary material). When all putative mediators (depression, impulsivity, and mood instability) were entered together, the effect comprised 38% of the total for suicidal attempts, and 38% for suicidal thoughts. Assessing the impact of individual mediators in separate analyses of suicide attempts, depression accounted for 23% of the total effect, impulsivity for 10% and mood instability 24%. The equivalent values in relation to suicidal ideation were almost identical at 23%, 9% and 23%. In the model linking *auditory hallucinations* with suicidal phenomena, the indirect and direct components were again positive and significant (Table 2b in supplementary material). The combined effect of the mediators explained 44% of the effect on suicide attempts and 36% of that on suicidal thoughts. The pattern of results for the individual mediators was similar to that for persecutory ideation: in relation to suicide attempts, depression accounted for 31% of the total effect, impulsivity for 10% and mood instability 26%. The corresponding figures for suicidal thinking were again very similar at 27%, 8% and 22%.

These results followed the same pattern in the APMS 2014 dataset (Tables 3a and 3b in supplementary material), although the effect of each component was less, in particular that of impulsivity. Thus for *persecutory ideation,* the overall effect of depression, impulsivity and mood instability combined was 24% for suicide attempts and 28% for suicidal thoughts. For suicide attempts, depression explained 19% of the effect, impulsivity 1% and mood instability 8%. For suicidal thoughts, depression explained 21%, impulsivity 2%, and mood instability 11%. Results for the model relating to *auditory hallucinations* and suicidal attempts and thoughts were very similar to those for persecutory ideation in the 2014 dataset. The combined effect of the mediators explained 23% of the effect on suicide attempts and 27% of that on suicidal thoughts. In relation to suicide attempts, depression accounted for 20% of the total effect, impulsivity for 2% and mood instability 7%. The corresponding figures for suicidal thinking were again very similar at 22%, 3% and 10%.

## 4 Discussion

### 4.1 Main findings

We sought to increase understanding of the impact of specific positive psychotic phenomena on the risk of suicidal thoughts and attempts, given its clinical and scientific importance. We undertook cross-sectional, longitudinal, and mediational analyses, using data from three British national psychiatric morbidity surveys using essentially identical methods. Replication of the analysis in separate datasets strengthens the validity of our findings.

In all datasets positive psychotic symptoms (persecutory ideas and auditory hallucinations) and probable psychosis were each cross-sectionally associated with lifetime suicide attempts and suicidal thoughts, even after controlling for confounders. The only exception was that persecutory ideation was not associated with suicidal attempts in the 2014 dataset.

Baseline persecutory thoughts also were significantly longitudinally associated with new inceptions of suicidal *ideation* at follow-up on an unadjusted level, but not repetition, or new inceptions of suicidal *attempts*. Auditory hallucinations were not longitudinally associated with repetition or inceptions of suicidal thoughts or attempts. Thus in the longitudinal analyses, we failed to demonstrate a linear relationship between our potential risk factors and outcome.

In the multiple regression analyses for both 2000 and 2007 datasets, auditory hallucinations were strongly associated with suicidal attempts and thoughts: however, this effect was moderated by the addition of persecutory ideation. This result differed in the 2014 dataset, where auditory hallucinations and persecutory ideation were both independently associated.

We also examined the role of potential mediators (depression, impulsivity, and mood instability) in the significant associations between psychotic symptomatology and suicide attempts and thoughts. For persecutory ideation and auditory hallucinations, the strength of the mediators combined varied between 36% and 41% (2007), and 24% and 28% (2014), leaving room for an appreciable direct effect of positive symptoms on suicidal thoughts and attempts. Mood instability made a larger contribution than impulsivity to the mediation of the effect of persecutory ideation on suicidal phenomena, and was similar for auditory hallucinations. This pattern was consistent across 2007 and 2014 datasets, but the effect was lower overall in 2014. The consistently lower impact of impulsivity as a mediator for both thoughts and attempts is interesting: it may indicate that mood instability itself includes important elements of impulsivity (de Cates et al., 2019). As might be expected, we also confirmed that depression was an important mediator for suicide attempts and thoughts in persecutory ideation and auditory hallucinations.

### 4.2 Strengths and weaknesses of our paper

Our detailed investigation of positive psychotic symptoms and suicidal phenomena included cross-sectional, longitudinal and mediation analyses. We used three very similar datasets where the data had been obtained with the same questions and in similar populations. As we had access to data with a longitudinal component, we were able to identify potential sequential effects in terms of psychotic symptoms and the emergence and persistence of self-harm. However, our analyses of “probable psychosis” were relatively constrained by small numbers: 40 in the 2000 dataset, 60 in the 2007 dataset and 94 in the 2014 dataset.

Household surveys like APMS sample thousands of people and are thus inevitably limited to the use of self-report and single questions to assess some variables. The APMS does not include variables for factors such as insight or subjective reasons for self-harm. Similarly, follow up interviews are likely to be limited to reduced subsets of the original sample, as in the current study. Such follow-up subsets may not be completely representative of the whole dataset. While the narrowly defined psychotic variables PSQ3a and 5a have superior specificity and consequent clinical utility, the longitudinal results for these variables were unstable. For auditory hallucinations in particular we obtained very large odds ratios and broad confidence intervals. We therefore used the broader variables PSQ3 and 5 for these analyses, which inevitably provide less specificity.

### 4.3 Implications

Suicide is a significant threat in people with schizophrenia: they have a lifetime risk 20 times that of individuals without schizophrenia, a concern to patients, relatives, carers and health care professionals alike (Nordentoft et al., 2015). Self-harm in schizophrenia appears to be independent of genetic risk, implying a significant environmental contribution (Laursen et al., 2017); such influences might be elucidated by epidemiological analyses of relevant cohorts. In the two datasets analysed here, there was significant overlap in the identified risk factors for suicidal thoughts and attempts in psychosis. These were consistent with those previously identified in the schizophrenia spectrum psychosis literature (Fuller-Thomson and Hollister, 2016; Hawton et al., 2005; Hor and Taylor, 2010; Nordentoft et al., 2015). Among individuals reporting suicidal ideation at baseline in a large community survey from the United States, psychotic experiences increased the risk of a suicide attempt more than threefold during the subsequent 12-month period, even after adjustment for other mental illness (DeVylder et al., 2015).

Between the onset of psychotic symptoms in the community and a first presentation to mental health services with a psychotic episode, around 10% may have engaged in self-harm (Challis et al., 2013)^42^, while the risk of self-harm during seven years follow-up may also be 10% (Challis et al., 2013). Our finding that suicide attempts and thoughts were associated with positive psychotic symptoms in cross-sectional analyses is important. Previous evidence has suggested that negative symptoms increase the risk of suicide (Hawton et al., 2005) and self-harm, particularly after treatment (Challis et al., 2013), and that positive symptoms may not be involved (Challis et al., 2013); our findings indicate that positive symptoms are also important, requiring clinicians to evaluate them in understanding, assessing and managing the risk of self-harm. Our results are not fully consistent with a recent meta-analysis of longitudinal data relating to psychotic experiences and suicidal ideation and acts (Yates et al., 2019) – that is, we did not find that psychotic experiences increased the risk of future suicidal acts. This may relate to different methods of defining psychosis within the included studies compared to our probable psychosis variable, and also the fact that the included studies focused on adolescents and young adults (c.f. the APMS which includes all adult ages). This ability to assess associations of self-harm and psychotic symptoms within a broader age range is a strength of our paper. We also may be providing more nuanced data, in that we highlight the potentially greater importance of persecutory ideation in terms of future suicidal thoughts. This highlights the complexity of interacting variables in understanding risk of self-harm.

It has been shown previously that mood instability is important for the genesis and persistence of psychosis (Marwaha et al., 2014; Palmier-Claus et al., 2012). Our results also suggest it has a significant effect on suicide risk in the context of positive psychotic symptoms, and may therefore offer potential targets for intervention. It was of much greater significance than impulsivity in our mediation analysis. If individuals can be helped to regulate their mood effectively, they may be less likely to experience suicidal thinking alongside their persecutory ideas and auditory hallucinations. We also found depression to be independently significant in the development of self-harm in psychosis, consistent with emerging evidence (McGinty et al., 2018). In the general population, the risk of any self-harm is potentially three times greater in individuals who report psychotic experiences versus those who do not: accompanying depression accounted for some but not all of this risk (Honings et al., 2016). Participants from the Collaborative Psychiatric Epidemiology Surveys who reported psychotic experiences were particularly likely to acknowledge concurrent suicidal ideation and suicide attempts (DeVylder et al., 2015). Over 65% of these individuals met criteria for DSM-IV depression, anxiety or a substance misuse disorder.

While psychiatric admission may partly reduce the immediate risk of self-harm in people with schizophrenia, it also may indicate an increased likelihood of future self-harm: the extent to which it reduces long-term suicide risk therefore remains unclear (Ayesa-Arriola et al., 2015). Although this is probably due to disease severity rather than to direct causation, the humiliation and stigma felt by individuals subjected to involuntary admission may itself directly increase suicidality (Large et al., 2017). In a Danish cohort study, approximately one third of suicides in schizophrenia took place during admission, or in the first week after discharge, and accounted for almost 3% of all suicides in the general population (Nordentoft et al., 2015).

We found the association of suicidal thoughts and acts with psychotic symptoms was most apparent cross-sectionally: one interpretation of this is that self-harm occurs in the context of a disturbed mental state, here represented by current distressing psychotic symptoms. This requires further exploration, both epidemologically and clinically. While we found that self-harm in psychosis is strongly linked to depression and mood instability, the sequence of events and the direction of cause and effect remains unclear. Nonetheless, our results confirm the link between positive psychotic symptoms and suicidality, and therefore improving symptoms may offer an effective way of reducing suicidal risk.

Future work could include assessment of the two less common psychotic variables within the APMS relating to thought agency and delusional mood, and exploration of cross-sectional and longitudinal associations between these and self-harm thoughts and acts. Another interesting line of enquiry would be to examine psychotic experiences as potential predictors of transition from ideation to acts, and the relative strength of this relationship (Hielscher et al., 2020).

## Data availability

The 2000 and 2007 datasets involved in this research are freely available, and additional information can be found in the survey reports (McManus et al., 2009; Singleton and Lewis, 2003). 2014 dataset is not freely available but the full report is freely accessible on NHS.digital (Byron et al., 2016). Ethical approval was received for the original data collection.

## Role of the funding source

Angharad de Cates has her salary and research costs supported by a 10.13039/100010269Wellcome Trust Clinical Doctoral Fellowship (216430/Z/19/Z). The Wellcome Trust had no other contribution to the article.

## CRediT authorship contribution statement

AdeC and MB formulated the research question. AdeC, MB, GC, SM, and PB were involved in designing the study. GC, AdeC, SM and CH performed the analyses. AdeC, GC and MB wrote the first draft, and all authors contributed to revisions.

## Acknowledgements/funding

This work was partially supported by the 10.13039/100010269Wellcome Trust (AdeC, grant reference: 216430/Z/19/Z).

## Declaration of competing interest

The authors declare that they have no known competing financial interests or personal relationships that could have appeared to influence the work reported in this paper.

## Appendix A Supplementary data

Supplementary tables Image 1
