# Supplementary material for: Self-harm, suicidal ideation, and the positive symptoms of psychosis: Cross-sectional and prospective data from a national household survey
Source: Schizophr Res. 2021 Jul;233:80–8. doi: 10.1016/j.schres.2021.06.021 (PMC8388846; doi:10.1016/j.schres.2021.06.021)
Supplement: Supplementary file 1 — Supplementary tables [file mmc1.docx]

**Supplementary material**

Table 1: 2000, 2007 and 2014 APMS descriptives

|  | 2000 | 2007 | 2014 |
| --- | --- | --- | --- |
|  | N (%) | N (%) | N (%) |
| **Sex**  Female  Male | 4728 (55.10)  3852 (44.90) | 4206 (56.81)  3197 (43.19) | 4488 (59.48)  3058 (40.52) |
| **Marital Status**  Married  Not married | 4383 (51.08)  4197 (48.92) | 4133 (55.83)  3270 (44.17) | 3367 (44.62)  4179 (55.38) |
| **Employment Status**  Employed  Unemployed | 5270 (61.88)  3246 (38.12) | 3989 (53.88)  3414 (46.12) | 3996 (52.95)  3550 (47.05) |
| **Ethnicity**  White British  White Other  Black  Asian  Mixed | 8031 (93.60)  α  185 (2.17)  159 (1.85)  139 (1.62) | 6499 (88.39)  308 (4.19)  188 (2.50)  199 (2.71)  159 (2.16) | 6387 (84.64)  426 (5.65)  197 (2.61)  357 (4.73)  151 (2.00) |
| **Highest Educational Qualification**  Degree  Other Higher  Sixth Form/A-Levels  Secondary School/GCSEs  Foundation Level  Other Qualifications  No Qualifications | 1239 (14.55)  614 (7.21)  1129 (13.26)  2968 (34.86)  940 (11.04)  α  2565 (30.12) | 1374 (18.99)  542 (7.49)  938 (12.96)  1817 (25.11)  α  286 (3.95)  2278 (31.49) | 1792 (23.75)  555 (7.35)  1184 (15.69)  1336 (17.70)  338 (4.48)  563 (7.46)  175 (2.32) |
| **Age Band**  75+  65-74  55-64  45-54  35-44  25-34  16-24 | ----------------  1268 (14.78)  1442 (16.81)  1545 (18.01)  1848 (21.54)  1683 (19.62)  794 (9.25) | 950 (12.83)  1028 (13.89)  1279 (17.28)  1130 (15.26)  1413 (19.09)  1035 (13.98)  568 (7.67) | 1062 (14.07)  1189 (15.77)  1226 (16.25)  1294 (17.15)  1180 (15.64)  1035 (13.71)  560 (7.42) |
| **IQ**  70-89 *(70-85)*  90-109 *(86-100)*  110-129 *(101+)* | 1620 (20.11)  3809 (47.29)  2625 (32.59) | 1053 (15.32)  1867 (27.17)  3952 (57.51) | 1094 (15.91)  1740 (25.30)  4043 (58.79) |
| **Sexual Abuse**  No  Yes | 8234 (95.97)  346 (4.03) | 6971 (94.75)  386 (5.25) | 7067 (93.99)  452 (6.01) |
| **Narrow persecutory symptoms**  No  Yes | 7771 (90.6)  809 (9.4) | 6834 (92.3)  569 (7.7) | 6958 (92.22)  587 (7.78) |
| **Narrow hallucinations**  No  Yes | 8498 (99)  82 (1) | 7335 (99.1)  68 (0.9) | 7445 (98.69)  99 (1.31) |
| **Broad persecutory symptoms**  No  Yes | 6767 (78.90)  1810 (21.10) | 6098 (82.43)  1299 (17.56) | 6234 (82.62)  1311 (17.38) |
| **Broad hallucinations**  No  Yes | 8203 (95.67)  371 (4.33) | 7074 (95.63)  323 (4.36) | 7198 (95.41)  346 (4.59) |
| **Probable Psychosis**  No  Yes | 8520 (99.30)  60 (0.70) | 7363 (99.46)  40 (0.54) | 7452 (98.75)  94 (1.25) |
| **Impulsivity**  No  Yes | 4673 (55.71)  3715 (44.29) | 4954 (68.68)  2259 (31.32) | 6027 (79.87)  1519 (20.13) |
| **Mood Instability**  No  Yes | 7195 (85.78)  1193 (14.22) | 6311 (86.11)  1018 (13.89) | 6690 (88.66)  856 (11.34) |
| **Depression Score**  0  1  2  3  4 | 6751 (78.68)  827 (9.64)  564 (6.57)  350 (4.08)  88 (1.03) | 5875 (79.36)  625 (8.44)  499 (6.74)  317 (4.28)  87 (1.18) | 6089 (80.69)  566 (7.50)  483 (6.40)  322 (4.27)  86 (1.14) |
| **Drug Misuse**  No  Yes | 6453 (75.54)  2089 (24.46) | 5720 (77.75)  1637 (22.25) | 5266 (74.33)  1819 (25.67) |
| **Alcohol Problems**  No  Yes | 6463 (75.70)  2075 (24.30) | 5789 (78.31)  1603 (21.69) | 5965 (82.64)  1253 (17.36) |
| **Suicide attempt in lifetime**  No  Yes | 8141 (94.98)  430 (5.02) | 7008 (94.77)  387 (5.23) | 7088 (94.08)  446 (5.92) |
| **Suicide thought in lifetime**  No  Yes | 7192 (83.90)  1380 (16.10) | 6311 (85.41)  1078 (14.58) | 6211 (82.41)  1326 (17.59) |
| **Had Suicidal Attempts since T1**  No  Yes | 2384 (99.09)  22 (0.91) | --------------  -------------- | --------------  -------------- |
| **T1 Suicidal attempts**  No  Yes | 2212 (92.05)  191 (7.95) | --------------  -------------- | --------------  -------------- |
| **Had Suicidal Thoughts sinceT1**  No  Yes | 2197 (91.31)  209 (8.69) | --------------  -------------- | --------------  -------------- |
| **T1 Suicidal thoughts**  No  Yes | 1820 (75.71)  584 (24.29) | --------------  -------------- | --------------  -------------- |

α = variable not present / conflated with nearest neighbour in this dataset

Table 2a: 2007 Mediation analyses between persecutory ideation (IV) and 1) suicidal attempts, 2) suicidal thoughts (DVs); mediators: depression, impulsivity, mood instability

| PERSECUTORY IDEATION (psq3a) | SUICIDE ATTEMPTS | SUICIDE THOUGHTS |
| --- | --- | --- |
| OR (CI)  *p *(<0.001), ** (<0.01), # (<0.05)* | | |
| Depression+impulsivity+mood instability  Total  Direct  Indirect | 6.32 (4.57-8.74)*  3.13 (2.25-4.34)*  2.01 (1.76-2.30)*  **38.11%** | 6.58 (5.20-8.32)*  3.20 (2.53-4.05)*  2.05 (1.82-2.30)*  **38.20%** |
| Depression  Total  Direct  Indirect | 6.23 (4.60-8.43)*  4.06 (2.99-5.50)*  1.53 (1.38-1.69)*  **23.38%** | 6.35 (5.10-7.91)*  4.13 (3.32-5.14)*  1.53 (1.41-1.67)*  **23.25%** |
| Impulsivity  Total  Direct  Indirect | 6.18 (4.58-8.33)*  5.19 (3.83-7.02)*  1.19 (1.11-1.27)*  **9.61%** | 5.77 (4.67-7.14)*  4.93 (3.99-6.09)*  1.17 (1.11-1.23)*  **9.03%** |
| Mood instability  Total  Direct  Indirect | 6.10 (4.46-8.35)*  3.98 (2.88-5.52)*  1.53 (1.37-1.69)*  **23.53%** | 6.37 (5.08-8.00)*  4.15 (3.31-5.21)*  1.53 (1.40-1.67)*  **23.11%** |

Table 2b: 2007 Mediation analyses between auditory hallucinations (IV) and 1) suicidal attempts, 2) suicidal thoughts (DVs); mediators: depression, impulsivity, mood instability

| AUDITORY HALLUCINATIONS (psq5a) | SUICIDE ATTEMPTS | SUICIDE THOUGHTS |
| --- | --- | --- |
| OR (CI)  *p *(<0.001), ** (<0.01), # (<0.05)* | | |
| Depression+impulsivity+mood instability  Total  Direct  Indirect | 11.04 (4.92-24.77)*  3.85 (1.67-8.83)*  2.86 (2.06-3.98)*  **43.87%** | 17.59 (7.98-38.75)*  6.25 (2.83-13.77)*  2.81 (2.03-3.88)*  **36.09%** |
| Depression  Total  Direct  Indirect | 11.87 (5.83-24.13)*  5.47 (2.66-11.23)*  2.16 (1.69-2.77)*  **31.31%** | 16.16 (7.92-32.96)*  7.67 (3.76-15.63)*  2.10 (1.67-2.65)*  **26.78%** |
| Impulsivity  Total  Direct  Indirect | 9.74 (5.02-18.90)*  7.80 (4.01-15.17)*  1.24 (1.07-1.44)*  **9.72%** | 12.12 (6.38-28.05)*  9.99 (5.27-18.93)*  1.21 (1.07-1.37)*  **7.77%** |
| Mood instability  Total  Direct  Indirect | 11.07 (5.13-23.87)*  5.91 (2.72-12.83)*  1.87 (1.42-2.46)*  **26.06%** | 16.70 (7.82-35.66)*  9.03 (4.24-19.23)*  1.84 (1.42-2.40)*  **21.84%** |

Table 3a: 2014 Mediation analyses between persecutory ideation (IV) and 1) suicidal attempts, 2) suicidal thoughts (DVs); mediators: depression, impulsivity, mood instability

| PERSECUTORY IDEATION (psq3a) | SUICIDE ATTEMPTS | SUICIDE THOUGHTS |
| --- | --- | --- |
| OR (CI)  *p *(<0.001), ** (<0.01), # (<0.05)* | | |
| Depression+impulsivity+mood instability  Total  Direct  Indirect | 7.05 (5.94-8.51)*  5.30 (4.50-6.36)*  3.22 (3.08-3.39)*  **23.56%** | 7.12 (6.27-8.30)*  5.09 (4.56-5.74)*  3.36 (3.22-3.50)*  **28.20%** |
| Depression  Total  Direct  Indirect | 7.04 (5.93-8.50)*  5.57 (4.75-6.65)*  3.12 (3.01-3.23)*  **19.10%** | 7.05 (6.17-8.11)*  5.43 (4.85-6.13)*  3.17 (3.08-3.27)*  **21.45%** |
| Impulsivity  Total  Direct  Indirect | 7.32 (6.20-8.78)*  7.18 (6.08-8.62)*  2.75 (2.71-2.78)  **1.42%** | 6.70 (5.94-7.62)*  6.57 (5.84-7.47)*  2.75 (2.72-2.77)#  **1.53%** |
| Mood instability  Total  Direct  Indirect | 7.24 (6.11-8.72)*  6.50 (5.37-8.04)*  2.88 (2.75-3.02)#  **8.20%** | 6.74 (5.96-7.69)*  5.93 (5.26-6.76)*  2.92 (3.11-3.03)*  **10.72%** |

Table 3b: 2014 Mediation analyses between auditory hallucinations (IV) and 1) suicidal attempts, 2) suicidal thoughts (DVs); mediators: depression, impulsivity, mood instability

| AUDITORY HALLUCINATIONS (psq5a) | SUICIDE ATTEMPTS | SUICIDE THOUGHTS |
| --- | --- | --- |
| OR (CI)  *p *(<0.001), ** (<0.01), # (<0.05)* | | |
| Depression+impulsivity+mood instability  Total  Direct  Indirect | 10.10 (7.29-14.74)*  6.71 (5.10-9.26)*  3.37 (3.13-3.65)*  **23.16%** | 8.78 (6.71-11.94)*  5.79 (4.66-7.41)*  3.45 (3.17-3.77)*  **27.47%** |
| Depression  Total  Direct  Indirect | 10.16 (7.38-14.71)*  7.12 (5.40-9.84)*  3.26 (3.07-3.46)*  **19.75%** | 8.66 (6.65-11.69)*  6.17 (4.94-7.94)*  3.27 (3.09-3.47)*  **22.19%** |
| Impulsivity  Total  Direct  Indirect | 9.81 (7.44-13.45)*  9.41 (7.14-12.89)*  2.77 (2.69-2.86)  **2.25%** | 7.59 (6.11-9.67)*  7.32 (5.92-9.27)*  2.77 (2.70-2.84)  **2.61%** |
| Mood instability  Total  Direct  Indirect | 9.16 (6.92-12.64)*  8.06 (6.07-11.20)*  2.89 (2.69-3.11)  **7.47%** | 7.52 (5.96-9.76)*  6.58 (5.28-8.43)*  2.91 (2.71-3.16)  **9.72%** |
